# Highly Stretchable, Transparent and Adhesive Ionogel Based on Chitosan-Poly(acrylic acid) Double Networks for Flexible Strain Sensors

**DOI:** 10.3390/gels8120797

**Published:** 2022-12-05

**Authors:** Yuan Zhu, Xuemei Li, Zhenjie Zhao, Yongri Liang, Limin Wang, Yingdan Liu

**Affiliations:** State Key Lab of Metastable Materials Science and Technology, College of Materials Science and Engineering, Yanshan University, Qinhuangdao 066004, China

**Keywords:** ionogel, double network, chitosan, adhesive, strain sensor

## Abstract

A stretchable double-network (DN) ionogel composed of a physically crosslinked network of chitosan (CS) and a chemically crosslinked network of polyacrylic acid (PAA) was prepared in an ionic liquid ([EMIM][OAc]) using a one-step polymerization method. In this ionogel (CS/PAA), the CS and the PAA polymer chains served as backbones, which constructed an interpenetrating DN structure via numerous hydrogen bonds formed through the hydroxyl, amino and carboxyl groups on the polymer chains. The DN structure improves the mechanical properties of the ionogel. Therefore, the CS/PAA DN ionogel exhibited outstanding mechanical performance in many ways: tensile strength up to 2.04 MPa, strain range up to 1046% and the value of toughness up to 8.52 MJ/m^3^. The ionogel also showed good self-recovery performance, fatigue resistance, ability to work in a broad temperature range (−20~80 °C) and adhesion properties. As a flexible sensor, the CS/PAA DN ionogel showed high strain sensitivity (gauge factor = 6.235). It can sensitively detect human motion (such as joint-bending, vocal fold vibration, walking gait and other human body motions), revealing the practical application potential of flexible electronic devices.

## 1. Introduction

The emergence of stretchable conductive materials has promoted the rise and development of flexible electronics, such as human motion detection devices [1], skin sensors [2], energy collection devices [3], stretchable batteries [4,5], stretchable electroluminescent devices [6,7,8] and stretchable touch panels [9,10]. Among different flexible electronics, skin-inspired wearable sensors can imitate the perception characteristics of human skin via transforming various external stimuli such as strain, pressure and temperature into electrical response signals. Therefore, this kind of sensor has promising applications in health monitoring [11,12,13], soft robotics [14,15,16], human–machine interface [17,18] and other emerging fields. Skin-inspired flexible wearable sensors can generally be divided into two categories: electronic skins (e-skins) [19,20] and ionic skins (i-skins) [21,22,23]. E-skins transduce electrical response signals via electronic conduction and are fabricated by embedding conductive nanofillers such as carbon nanotubes (CNTs) [24], graphene [25], silver nanowires (AgNWs) [26] and MXene [27] in an insulating polymer matrix as a permeable conductive filler network. Nevertheless, fillers with poor electrical conductivity cannot ensure the formation of a permeable conductive path, and the increase in conductive fillers inevitably leads to an increase in the modulus of the composite, resulting in a serious mismatch with the matrix during the deformation process [28]. The incompatibility between the conductive nanofillers and the elastomeric matrix produces agglomerates of nanofillers and severely restricts the movement of the polymer network during the deformation process [29]. In addition, the inherent opacity of conductive fillers also limits their applications that require transparency.

Unlike e-skins, which transmit electrical response signals via electronic conduction, i-skins simulate the sensing function of human skin via ionic conduction [21]. In general, i-skins can be prepared from stretchable and ionic conductive hydrogels and ionogels [30,31,32,33,34,35]. They are composed of polymer networks swollen by inorganic salt solutions and ionic liquids, respectively. Hydrogels can be used as an ideal ionic skin because of their good stretchability, transparency and sensitivity. However, there are still a lot of issues that need to be solved for hydrogels in terms of cryogenic freezing, evaporation of water at high temperature and being non-conductive for long-term storage in practical applications of hydrogels [36,37]. Moreover, hydrogels have low adhesion strength and do not adhere well to the surfaces of other materials [38]. Adding salts to hydrogels can improve the water retention capability of hydrogels [39]; however, it cannot stop the evaporation of water at high temperature. Dielectric elastomer encapsulating is another method of enhancing the stability of hydrogels under high humidity and high temperature [40]. Nevertheless, it brings another problem; that is, the motion of the human body is obstructed, which further leads to poor electromechanical properties. Additionally, this method has a complex manufacturing process and limits the enhancement of stability. Introducing organic solvents (such as dimethyl sulfoxide [41] and glycerol [42]), ionic liquids [31] and inorganic salts [43] are three extensively used methods for restraining the production of ice crystals and improving anti-freezing ability. The component of free water can be changed by adding ions or replacing it with anti-freezing solvents. Therefore, the water content can be decreased to reduce the freezing temperature of the hydrogels. Nevertheless, the unknown biocompatibility performance of some organic solvents has a passive impact on multifaceted properties of hydrogels [44]. Moreover, encapsulation and plasma etching of hydrogels, which have tedious steps, are required to protect against the leakage of liquid solvents. Furthermore, too much salt damages the network structure of hydrogels, resulting in a decrease in mechanical properties [45].

Ionogels show unique properties because of the combination of the characteristics of ionic liquids and polymer networks. In general, 3D polymer networks bring ionogels high flexibility, outstanding mechanical properties and certain integrity. At the same time, ionic liquids show negligible vapor pressure, excellent ionic conductivity, nonflammability and thermal/chemical/electrochemical durability, and possess interactions with polymer chains. Therefore, ionogels are good alternatives to hydrogels [30,31] that can work under extreme environments and have shown great potential in the field of flexible wearable sensors. However, ionogel-based sensors generally have low stretchability, mechanical properties and fatigue resistance, and are prone to fracture during deformation, which severely hinder their applications in flexible wearable sensors. It is a valid means to enhance the mechanical performance of ionogels by constructing double network (DN) architecture. Kamio et al. reported that a rough DN ionogel consisted of silica particles and an interpenetrating network of organic polymer poly(dimethyl acrylamide) [46]. The DN ionogel showed extremely high mechanical strength (compressive fracture strength over 28 MPa) at ionic liquid content of 80 wt%. Due to the high stretchable properties of physical crosslinked ionogels reported at present, they usually showed bad elastic recovery from large tensile deformations, poor reliability and durability [28,47]. On the contrary, chemical crosslinked ionogels with chemical covalent bonds exhibit outstanding elastic recovery from large tensile deformations and good reliability and durability, but the problems are low break elongation and poor toughness [31,48]. A practicable method for the synthesis of ionogels with high stretchability, mechanical strength and fatigue resistance is to construct a DN structure with both a physically crosslinked network (sacrificial network) and chemically crosslinked network (covalent network). Moreover, the ionogel sensors need strong adhesion with the substrate to obtain jar-less and credible signals [23]. However, ionic liquids generally lack adequate interfacial adhesion with substrates. Therefore, the fabrication of an ionogel with good adhesion properties has become an important challenge.

To solve the above problems, we designed and fabricated an ionogel with excellent mechanical properties and strain sensitivity. The ionogel consisted of a physically cross-linked network of chitosan (CS) and a chemically cross-linked network of poly(acrylic acid) (PAA), in which numerous hydrogen bonds were formed between the CS network and PAA network. The introduction of CS not only greatly improved the adhesion performance, but also decreased the risk of chemical deleterious effects and immune response on human body. The CS/PAA DN ionogel showed high mechanical properties (tensile strength up to 2.04 MPa when the elongation strain achieved 1046%), high transparency (88%), good self-recovery and fatigue resistance. Because the ionogel contained a large amount of ionic liquid, it presented high ionic conductivity, good toughness and resilience over a wide temperature range (−20~80 °C). In addition, as a kind of flexible sensor, the strain sensitivity of the ionogel (gauge factor (GF) value is 6.235) is high, which can sensitively detect human activities (such as joint-bending, vocalization, walking gait and other human body motions) with rapid response and good repeatability.

## 2. Results and Discussion

### 2.1. Construction of CS/PAA DN Ionogel

**Scheme 1:**
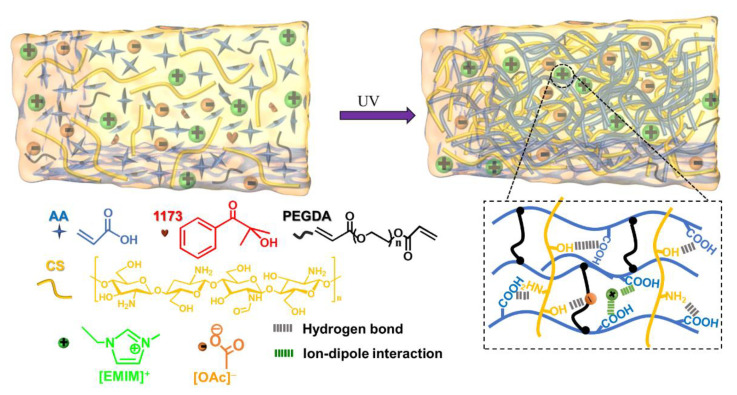
Schematic diagram of the preparation of CS/PAA DN ionogel.

As illustrated in Scheme 1, the mixed solution includes CS chains, monomer AA, [EMIM][OAc], photo-initiator Irgacure 1173 and crosslinking agent PEGDA. The polymerization was initiated under the influence of UV light to form a crosslinked PAA network, in which CS chains are interpenetrated and interact with PAA via the hydrogen bond formed between -OH/-NH_2_ and -COOH. Therefore, the CS/PAA DN ionogel combines physical crosslinking of weak hydrogen bonds and strong chemical crosslinking of covalent bonds together. Numerous hydrogen bonds formed among CS, PAA and ionic liquid can dissipate a lot of energy when the ionogel is stretched. In addition, ion-dipole interaction exists between the ionic liquid and the carboxyl groups of PAA chains, which helps to improve the compatibility between ionic liquid and the polymer chains and enhance the stability of the ionogel.

**Figure 1:**
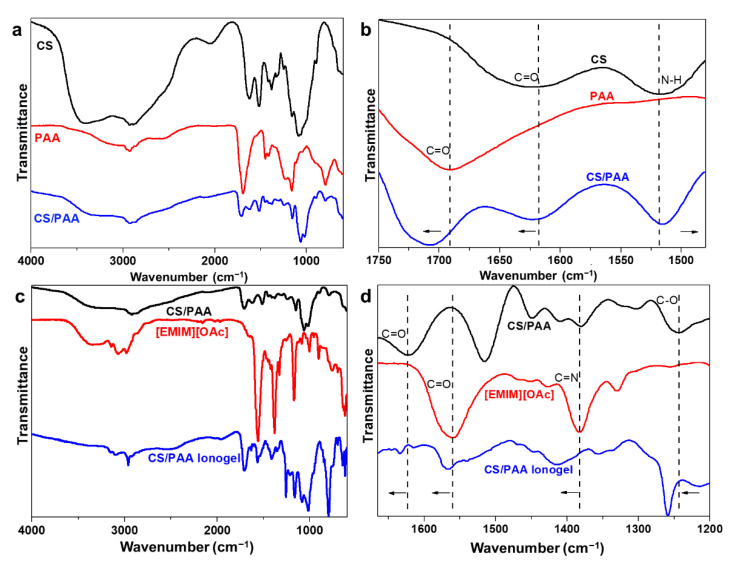
FTIR spectra of CS, PAA, and dried CS/PAA networks in a broad wavelength range of 4000–400 cm^−1^ (**a**) and a selected range of 1750–1480 cm^−1^ (**b**); FTIR spectra of ionic liquid [EMIM][OAc], dried CS/PAA networks and CS/PAA DN ionogel in a broad wavelength range of 4000–400 cm^−1^ (**c**) and a selected range of 1665–1210 cm^−1^ (**d**).

The existence of physical interactions (hydrogen bond and ion–dipole interaction) can be verified from the FTIR spectra. As shown in Figure 1a,b, the transmission peaks of carbonyl group C=O and of the N-H bond in the CS, respectively, appear at 1618 cm^−1^ and 1518 cm^−1^. The transmission peaks of carbonyl group C=O of PAA appears at 1691 cm^−1^. To detect the interaction between CS and PAA, the FTIR spectrum of a dried CS/PAA network was analyzed and compared with that of pure CS and PAA. The peak positions of the above three vibrations of CS and PAA moved slightly, indicating that a hydrogen bond formed between CS and PAA. In Figure 1c,d, the transmission peaks of the carbonyl group of CS in the dried CS/PAA network and the transmission peaks of the C-O bond in PAA separately appears at 1623 cm^−1^ and 1243 cm^−1^. The transmission peaks of carbonyl group C=O and of the C=N bond of [EMIM][OAc] separately appears at 1560 cm^−1^ and 1382 cm^−1^. For the CS/PAA DN ionogel, which contains both the CS/PAA network and [EMIM][OAc], the above four characteristic peaks shifted slightly, indicating that a hydrogen bond and ion–dipole interaction formed between the polymer chains and [EMIM][OAc]. To confirm the percentage of hydrogen-bonded functional groups, the FTIR spectra of CS/PA ionogel in a specific range was analyzed using a curve-fitting process. As shown in Figure S1, both free N-H/C=O and hydrogen-bonded N-H/C=O stretching are involved in the absorption peak. The percentage of the hydrogen-bonded groups can be calculated from the peak area ratio (Table S1). The percentage of hydrogen-bonded C=O groups of CS is 72%, and is 56% for PAA in the CS/PAA ionogel.

**Figure 2:**
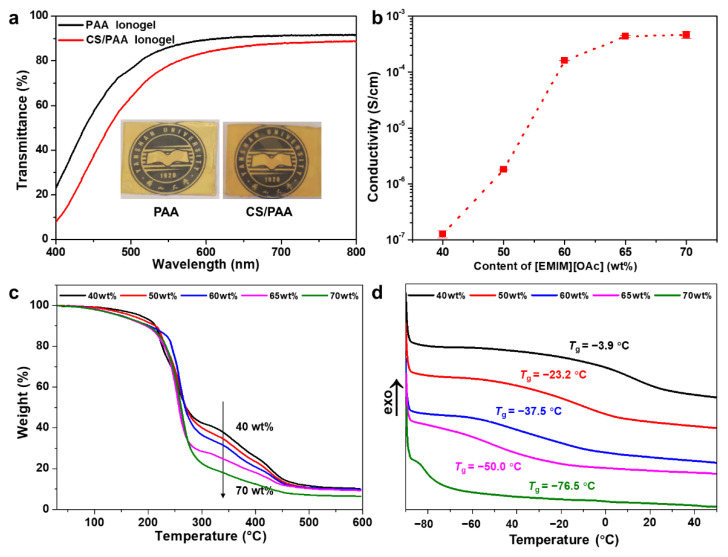
Transmittance of PAA and CS/PAA DN ionogels (**a**), conductivity (**b**), TGA (**c**) and DSC curves (**d**) of the ionogels with different contents of ionic liquid.

Due to the good compatibility between PAA and [EMIM][OAc], PAA ionogel has high transparency. As shown in Figure 2a, the transmittance of the PAA ionogel in the visible region reaches 91%. The addition of CS reduces the transparency of the ionogel slightly, the transmittance of which is about 88%. The CA/PAA ionogels also have good ionic conductivity. Figure 2b depicts the relationship between conductivity of the ionogels and ionic liquid content, which are measured at 25 °C. The conductivity of ionogel rose significantly from 10^−7^ S/cm to 10^−4^ S/cm with the increase in ionic liquid content from 40 wt% to 60 wt%. When the content of ionic liquid increases continuously to 70 wt%, the conductivity of the ionogel does not change a lot as shown in the range of 60–70 wt%. Therefore, 60 wt% of ionic liquid is the critical content in the ionogel. Different from hydrogels, in which water is volatile even at room temperature, ionogels have excellent thermal stability. As shown in Figure 2c, the weight of ionogel decreased significantly until 200 °C, indicating that the ionogel has high thermal stability. The glass transition temperature (*T*_g_) of the ionogels was measured by DSC. As shown in Figure 2d, there is only one obvious *T*_g_ for each ionogel, which proves that the ionogels have uniform structures without phase separation. Since the *T*_g_ of [EMIM][OAc] is extremely low (−78 °C) [49], *T*_g_ of the ionogel decreases as the [EMIM][OAc] content increases. As the content of [EMIM][OAc] increases from 40 wt% to 70 wt%, the *T*_g_ value decreases from −3.9 to −76.5 °C. The low *T*_g_ makes the ionogels flexible at very low temperature.

### 2.2. Adhesive Properties

**Figure 3:**
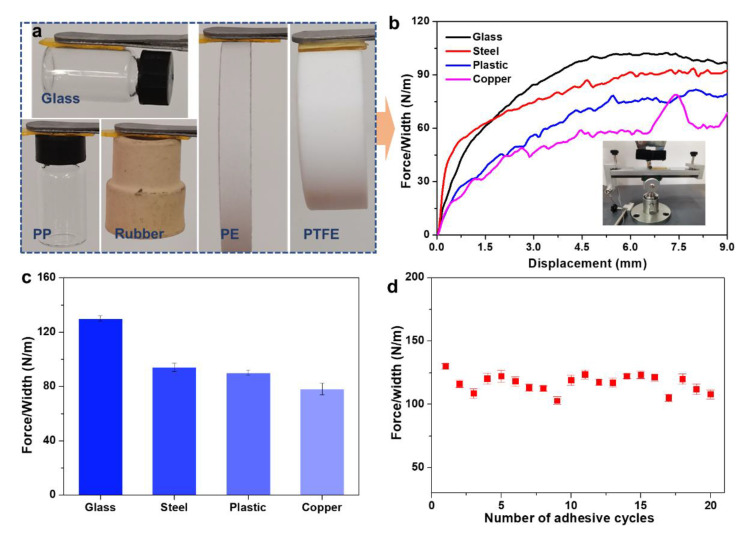
Optical images of adhesion of the CS/PAA DN ionogel to different materials (glass, PP, PE, rubber and PTFE) (**a**); force-displacement curve (**b**) and adhesion force (**c**) to different materials measured by 90° peeling tests; adhesion force of the CS/PAA DN ionogel to glass slide measured by 90° peeling test for 20 cycles (**d**).

Figure 3a–d shows the adhesive performance of the CS/PAA DN ionogel with various materials. As shown in Figure 3a, the solid substrate materials including glass, PP, PE, rubber and PTFE can be adhered onto the CS/PAA DN ionogel and lifted by adhesion force without falling. The good adhesive properties of the ionogel are mainly due to the polar groups of -OH, -COOH and -NH_2_, which can form hydrogen bond interactions, metal coordination interactions, ion-dipole interactions and other non-covalent interactions with the surface of various solid materials. The adhesion of ionogel to various materials is beneficial to combining the ionogels with other components in practical use.

The adhesive properties of CS/PAA DN ionogel were quantitatively measured by 90° peeling tests. The inset of Figure 3b shows the 90° peeling fixture of the tensile machine. The ionogel was adhered on the horizontal substrate with one side clamped by the moving part of the tensile machine. The peel strength curves of the ionogel on solid substrates such as glass, stainless steel, plastic (PE) and copper are shown in Figure 3b, and the adhesive strengths are 130.1 N/m, 94.1 N/m, 90.1 N/m and 78.7 N/m (Figure 3c), respectively. The results indicate that the ionogel has certain adhesion ability on different substrates. The repeated and persistent adhesion properties of the ionogel were studied by 20 successive adhesion/stripping cyclic tests. As shown in Figure 3d, the adhesion strength of the CS/PAA DN ionogel fluctuates in the range of 102–130 N/m, confirming its good adhesion performance. This repeated adhesion ability is greatly important for the extensive application of ionogel.

### 2.3. Mechanical Properties

**Figure 4:**
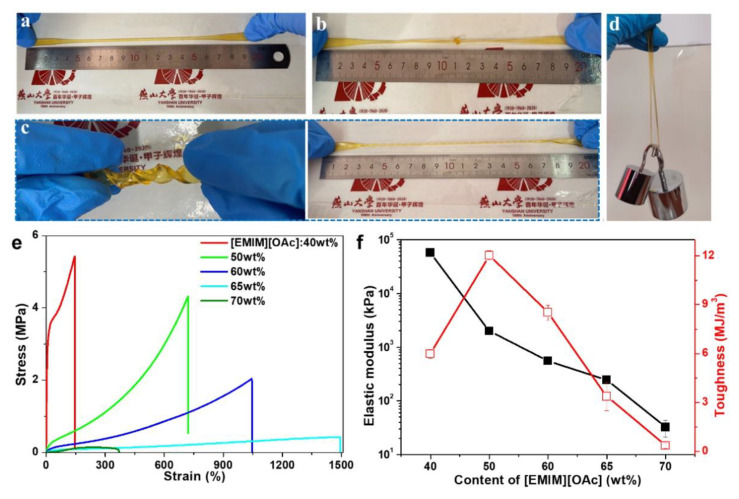
Excellent mechanical properties of the CS/PAA DN ionogel: tensile (**a**), knotted tensile (**b**), twisted tensile (**c**) and bearing a weight of 700 g with no damage (**d**); tensile performance of the ionogels with different contents of ionic liquid (**e**) and the corresponding elastic modulus (solid) and toughness value (open) (**f**).

The CS/PAA DN ionogel shows excellent toughness. As shown in Figure 4a–c, the ionogel can be stretched, knotted and twisted without fracture. The sample had an original size of 50 mm and could be stretched up to four times its original size. Figure 4d shows that the ionogel can retain up to 700 g of mass without breaking. Figure 4e,f shows the tensile stress–tensile strain curves of the ionogel at different ionic liquid contents and the values of elastic modulus and toughness. In an ionogel, ionic liquid plays the role of plasticizer. With the increase of ionic liquid [EMIM][OAc], the strength, modulus and toughness of the ionogel decrease, while the elongation at break increases (except for the sample containing 70 wt% of ionic liquid, the network of which is too thin to bear stretching). When the content of [EMIM][OAc] is 60 wt%, the ionogel has excellent mechanical properties. The tensile strength can reach 2.04 MPa with an elongation of 1046% and a toughness value of 8.52 MJ/m^3^. For further mechanical analysis, the ionogel sample with 60 wt% of [EMIM][OAc] was selected.

**Figure 5:**
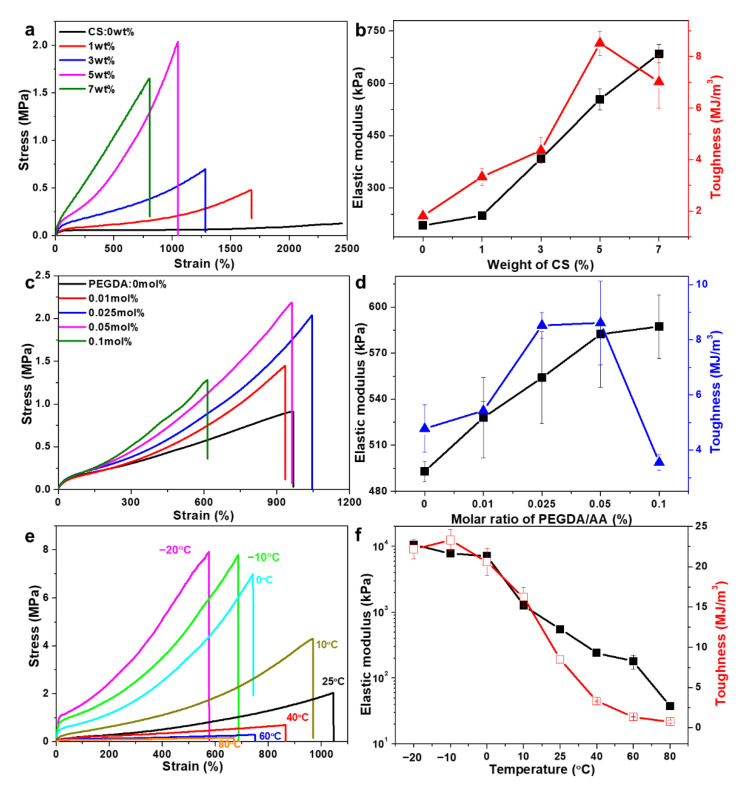
Tensile properties of the CS/PAA DN ionogels: tensile stress–tensile strain curves (**a**) and the corresponding elastic modulus (square) and toughness (triangle) values (**b**) of the ionogel with different contents of CS (weight fraction to AA); tensile stress–tensile strain curves (**c**) and the corresponding elastic modulus (square) and toughness (triangle) values (**d**) of the ionogel with different contents of PEGDA (molar fraction to AA); tensile stress–tensile strain curves (**e**) and the corresponding elastic modulus (solid) and toughness (open) values (**f**) of the ionogel measured at different temperatures.

In addition to the content of ionic liquid, the amount of CS and PEGDA also showed significant influence on the mechanical properties of the ionogel. The CS chains in the DN ionogel participated in the construction of internal network through hydrogen bond interaction and improved the mechanical properties of the ionogel. To verify the excellent mechanical performance of the ionogel, tensile stress–tensile strain tests were carried out. Figure 5a,b shows the tensile stress–tensile strain curves of the ionogel with different CS contents and the values of elastic modulus and toughness. With the increase in CS content, the modulus of ionogel increased and the elongation at break decreased. The highest tensile strength of 2.18 MPa was achieved when the CS content was 5 wt%, with a tensile elongation of 961% and a toughness value of 8.61 MJ/m^3^. Hence, for further mechanical analysis, the ionogel sample with 5 wt% of CS was selected. This is because when the CS content becomes higher, there are more polar groups to form hydrogen bonds between the polymer chains, which improves the crosslinking density of the polymer network and thus increases the tensile strength of the ionogel. Nevertheless, when the CS content reached 7 wt%, the strength and toughness values decreased. The reason is that the excessively stiff CS chains improved the brittleness and decreased the toughness of the ionogel. The content of crosslinking agent PEGDA also had an obvious impact on the mechanical performance of the ionogel. As shown in Figure 5c,d, the increase in PEGDA content greatly enhanced the mechanical performance of the ionogel. However, when the PEGDA content was too high, the tensile strength and the value of toughness were greatly reduced. This is due to the fact that the high crosslinking density restricts the slip motion of the polymer chains, resulting in stress concentration and less energy dissipation by breaking non-covalent bonds. The tensile strength of 2.04 MPa is achieved when the PEGDA content is 0.025 mol%, with a tensile elongation of 1046% and a toughness value of 8.52 MJ/m^3^. Therefore, for further mechanical analysis, the ionogel sample with 0.025 mol% of PEGDA was selected. 

The tensile properties of the ionogel were tested at different temperatures to verify the elasticity of the ionogel at high and low temperature. As shown in Figure 5e,f, the ionogel still presents good mechanical performance and flexibility in the low temperature range of −20 to 10 °C. Compared with its mechanical properties at room temperature, the ionogel shows higher tensile strength and lower elongation at low temperatures due to the weakened mobility of the polymer segment. However, the value of elongation is still higher than 500%. Even at −20 °C, the ionogel still presents good tensile properties with a tensile strength of 7.9 MPa and elongation at break of 578%. It confirms that ionogel has good elasticity and toughness at low temperature. At high temperature, the mobility of polymer segments increases, which destroys non-covalent interactions such as hydrogen bonds in the ionogel, leading to a decrease in the tensile strength of the ionogel even though, due to the high stability of the chemical crosslinking network of PAA, the mechanical performance of the CS/PAA DN ionogel at 80 °C is still at a normal level with an elastic modulus of 33 kPa, a tensile strength of 167 kPa and elongation at break of 670%. Therefore, the CS/PAA DN ionogel has good mechanical properties across a broad temperature range.

**Figure 6:**
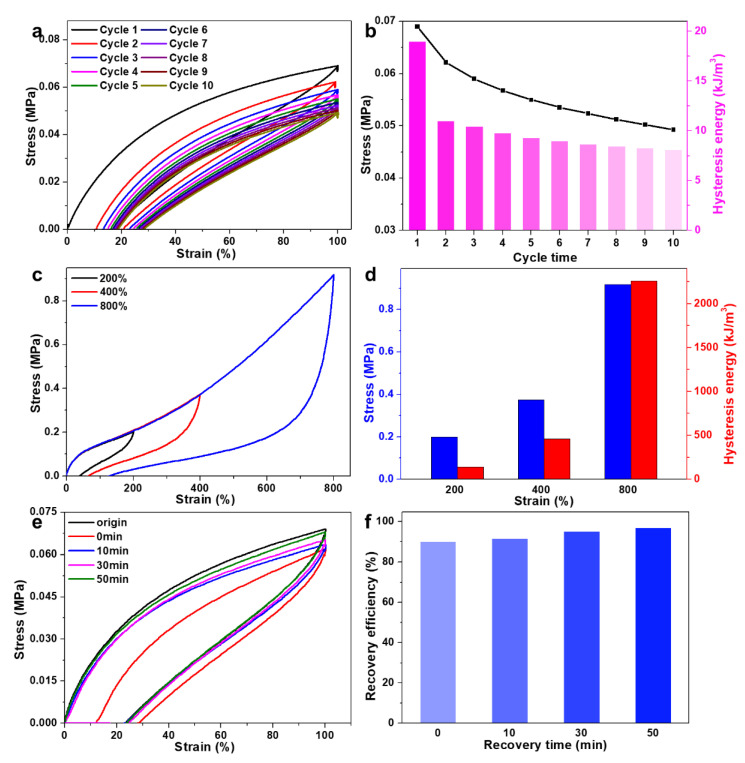
Anti-fatigue performance of the CS/PAA DN ionogel: cyclic tensile curves (**a**) and corresponding stress and dissipated energy values (**b**) for 10 consecutive cycles under a strain of 100%; cyclic tensile curves (**c**) and the value of stress and dissipated energy (**d**) under different maximum strain; cyclic tensile curves (**e**) and corresponding recovery efficiency (**f**) after different relaxation time.

Figure 6 shows the antifatigue performance of the CS/PAA DN ionogel. Figure 6a presents the tensile stress–tensile strain curves of 10 consecutive cycles of loading and unloading tensile tests under a fixed strain of 100% without rest. The corresponding maximum stress and toughness values are shown in Figure 6b. The first cyclic stretching test exhibits an obvious hysteresis loop and large amount of dissipated energy, indicating that many crosslinking points in the ionogel were destroyed by the first tensile test and a lot of energy was dissipated. The maximum stress and dissipation energy of the 2–10th cyclic curves decreased slightly, showing good repeatability. This indicates that the physical interactions such as hydrogen bonds and ion–dipole interactions in the network can be reversibly form after being broken, providing good self-recovery properties to the ionogel. As shown in Figure 6c, hysteresis loops and remanent strains are seen in the cyclic stretching curve at different maximum strains. The corresponding maximum stress and toughness values are shown in Figure 6d, both of which rise as the stretching strain increases. When the strain is 800%, the value of tensile stress reaches 0.92 MPa and the energy dissipation value reaches 2.26 MJ/m^3^. The appearance of lag rings is associated with local breaks in the abundance of non-covalent interactions in the ionogel. These results indicate that as the strain increases, more non-covalent bonds are broken in the ionogel network, which consumes more energy and causes more structural change. Under a fixed strain of 100%, experiments with different relaxation times (0–50 min) were carried out (Figure 6e). The recovery efficiency (RE) is evaluated as the specific value of the recovered tensile stress to the original tensile stress. It can be seen from Figure 6f that the ionogel can reach an RE of 91.5% after relaxation for 10 min after the first stretching. As the relaxation time increases, the recovery efficiency rises gradually. The RE can reach 97% when the relaxation time is 50 min. The reversible hydrogen bonds and ion–dipole interaction endow the ionogel with good self-recovery properties.

### 2.4. Strain-Sensitive Performance

**Figure 7:**
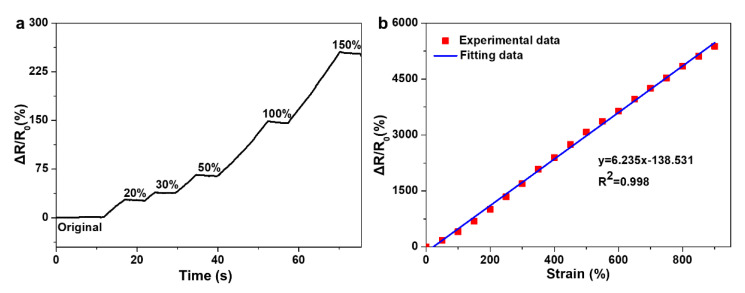
The relative resistance change of the CS/PAA DN ionogel with time (**a**) and the relative resistance change of the CS/PAA DN ionogel as a function of stretching strain (**b**).

The CS/PAA DN ionogel was used as a flexible strain sensor to investigate the relationship between the resistance or conductivity of the ionogel and tensile strain. As illustrated in Figure 7a, the relative resistance change (ΔR/R_0_) of the ionogel shows good response to tensile strain: the value of ΔR/R_0_ increased with strain and remained constant when the stain was fixed at a certain value. As shown in Figure 7b, when the strain is increased linearly, the value of ΔR/R_0_ also increases linearly with the strain. This is because during the stretching process, the length (L) of the ionogel increased and the cross-sectional area (S) of the ionogel reduced, leading to a reduction in ion concentration and lengthening of the pathway for ion transport. According to the formula for resistance calculation *R* = *L*/(*S* × *σ*), it can be seen that the resistance increases as the ionogel is stretched. Therefore, the ionogel can transform the mechanical deformation into an electrical signal, which can be used for the strain sensor. The relative resistance change curve can be well-fitted by using the formula: y = ax + b, in which y is the relative resistance change, x is the tensile strain, a and b are fitting parameters. The fitting result is a = 6.235 and b = −138.531, therefore, the sensitivity (GF value) of the ionogel is 6.235. The linear relationship between the resistance change and the tensile strain facilitated the calibration process, improving signal accuracy and reliability.

**Figure 8:**
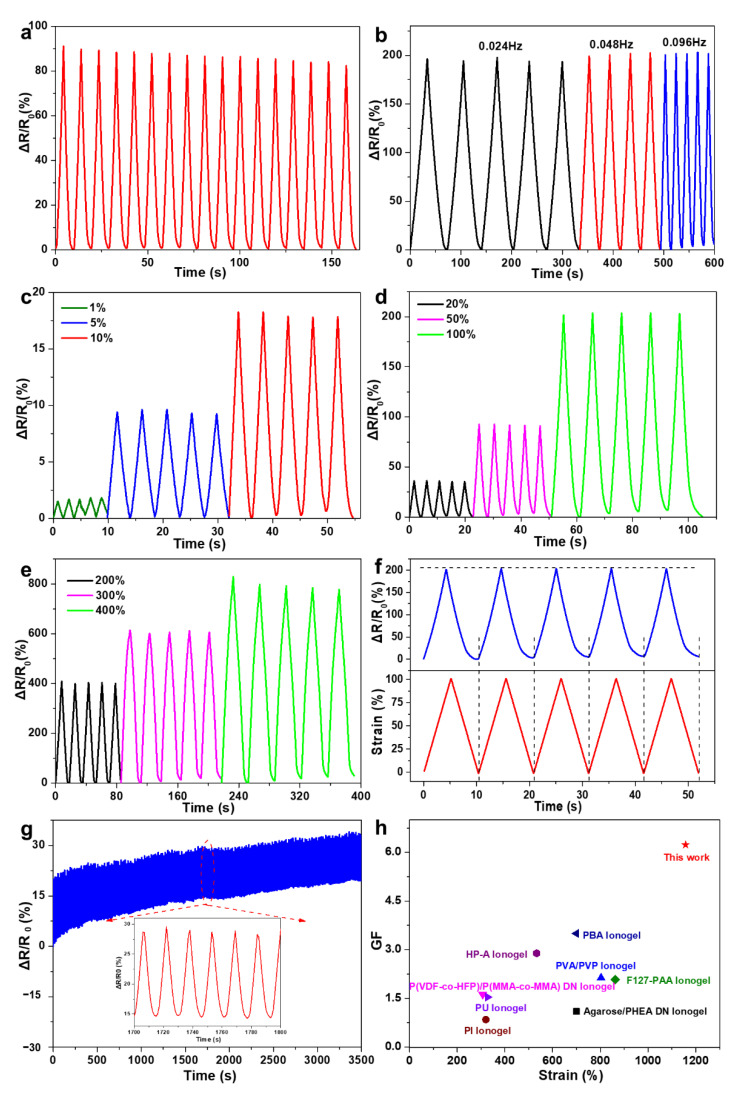
Sensing performance of the CS/PAA DN ionogel sensor: relative resistance change curve corresponding to 17 consecutive tensile cycles under a fixed strain of 50% (**a**); relative resistance change curve corresponding to different frequencies under a cyclic tensile strain of 100% (**b**); relative resistance change curve under different strain (**c**–**e**); the relationship between the input cyclic strain (100%) and the output signal of the relative resistance change (**f**); the relative resistance change curve corresponding to 200 continuous stretching cycles under a strain of 10% (**g**); the comparison of elongation strain and GF value of the reported ionogel flexible sensor (**h**): PI ionogel [50], Agarose/PHEA DN ionogel [51], PU ionogel [52], F127-PAA ionogel [53], P(VDF-co-HFP)/P(MMA-co-MMA) DN ionogel [54], PVA/PVP ionogel [55], PBA ionogel [48] and CS/PAA ionogel in this work.

Figure 8 shows the sensing performance of the CS/PAA DN ionogel under cyclic stretching. As shown in Figure 8a, the ionogel can maintain stable sensing properties over 17 consecutive stretching cycles. Figure 8b shows that the ionogel has the ability to identify stretching deformation at different frequencies. As the frequency increases, the peak of the relative resistance change becomes denser, which can reflect the change in frequency. It is of great significance to identify frequently changed motion or deformation in real-time monitoring. As shown in Figure 8c–e, at different stretching strains, the relative resistance change exhibits different values, and remains relatively stable and repeatable under the same strain. This indicates that the ionogel can respond not only to small strain (Figure 8c) and medium strain (Figure 8d), but also to large strain (Figure 8e), proving that the ionogel has good working reliability across a large strain range. Moreover, by comparing the ΔR/R_0_–time curve with the strain–time curve, it can be found that the trend of the ΔR/R_0_–time curve and the trend of the strain–time curve is the same in the process of stretching (Figure 8f), indicating that the ionogel can achieve real-time and rapid detection of the deformation signal. As shown in Figure 8g, after 200 cycles under a strain of 10%, although the electrical signal shifts with the increase of stretching times, the relative resistance change does not increase as the movement times increase, indicating that the ionogel sensor has durability and stability. The reason for the deviation in the electrical signal may be due to the fact that in the continuous stretching process, the reversible physical interaction destroyed for energy dissipation cannot be recovered immediately. As a result, the ion transport channel inside the ionogel cannot be recovered immediately after being damaged. As shown in Figure 8h, compared with other ionogels, the CS/PAA DN ionogel prepared in this work presents outstanding sensitivity and flexibility.

**Figure 9:**
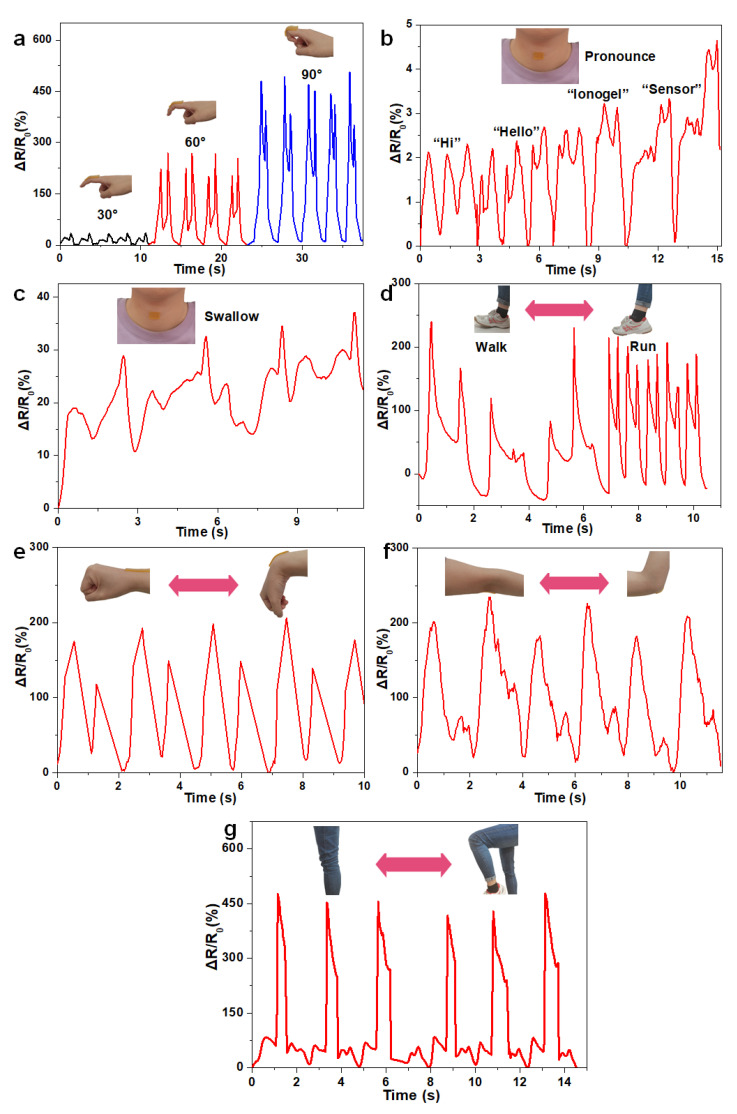
The CS/PAA DN ionogel as a flexible sensor to detect practical human physiological activity: continuously bending fingers at different angles (**a**), vocalization when saying some different words (**b**), throat swallow jitter (**c**), pressure on foot when walking and running on the ground (**d**), persistent bending joints from different parts of the body (wrist (**e**), elbow (**f**) and knee joint (**g**)).

Due to its good tensile properties, transparency, adhesive properties and strain sensitivity, the CS/PAA DN ionogel was applied as a flexible strain sensor, which can be directly adhered on the surface of human skin to monitor human activities through the relative resistance changes in real time. By sticking the ionogel to the finger, the ionogel sensor distinguished different finger bending angles during the test, and the electrical signal remained stable when the finger remained at the same bending angle (Figure 9a). The ionogel sensor can also identify small deformations such as vocal fold vibrations caused by speech. As shown in Figure 9b, the ionogel sensor detected subtle strain changes when volunteer said different words, which can be used for the purpose of speech recognition. The ionogel sensor also sensed the deformation of the throat when swallowing occurred (Figure 9c). When the ionogel sensor was attached to the bottom of the foot, it distinguished walking and running movements in real time (Figure 9d). In addition, the ionogel sensor was able to distinguish joint flexion in different parts of the body, including the wrist, elbow and knee (Figure 9e–g). Because the motion amplitude of each joint is different, the bending behavior of different joints can be distinguished by comparing the relative resistance change. This confirms that the ionogel sensor has high sensitivity and good repeatability, indicating potential applications in the area of flexible wearable devices.

## 3. Conclusions

In summary, we successfully prepared a CS/PAA double network ionogel with high transparency, high ionic conductivity, high mechanical properties, adhesion and wide operating temperature range. The strategy of constructing double-network ionogel by introducing a physically crosslinked CS network into a chemically crosslinked PAA network greatly improved the mechanical properties of the ionogel and afforded the ionogel good self-recovery and antifatigue properties. By varying the contents of ionic liquid, CS and PEGDA, the ionogel can achieve a high tensile strength up to 2.04 MPa and an elongation strain of 1046%. The application of the ionogel as a flexible strain sensor was possible because of its excellent electromechanical properties. The CS/PAA ionogel sensor not only showed high sensitivity (GF value up to 6.235), but also showed sensitive response to deformation across a wide strain range and at different frequencies. The CS/PAA ionogel sensor was successfully used to detect human body movement in different modes including bending of fingers and limbs, vocal cord vibration, and other delicate and complex movements of muscle. Its excellent mechanical and sensing performance allows the ionogel to have potential applications in flexible wearable devices.

## 4. Materials and Methods

### 4.1. Materials

Water-soluble chitosan (CS) (>90%, deacetylation) was purchased from Huantai Jinhu Shell Products Co. Ltd. Acrylic acid (AA) (99%) and poly(ethylene glycol) diacrylate (PEGDA, Mn = 400 g/mol) were supplied by Macklin. 2-Hydroxy-2-methylpropiophenone (Irgacure 1173, 98%) is a product of Aladdin. 1-ethyl-3-methylimidazolium acetate ([EMIM][OAc]) (99%) was supported by Lanzhou Greenchem ILs Co. Ltd. and dried at 120 °C for 6 h before use.

### 4.2. Fabrication of CS/PAA Ionogels

The first step was to dissolve the water-soluble CS in [EMIM][OAc] at 110 °C under an inert atmosphere of N_2_ with stirring. When the solution became transparent, monomer AA, Irgacure 1173, and crosslinking agent PEGDA were poured into the solution. Ultrasonication was applied to the solution to remove air bubbles, and then the solution was infused into a glass mold. Then, the glass mold was exposed to an ultraviolet light (365 nm, 6 W) for 4 h. Dosage of the chemicals for the different ionogel samples are shown in Table S2. Tensile strength, tensile elongation and toughness of different ionogel samples are shown in Table S3. 

### 4.3. Characterization

#### 4.3.1. Structures

FTIR spectroscopy of the samples was performed using a Thermo Scientific Nicolet iS20 spectrometer. The dried CS/PAA sample was prepared by replacing ionic liquid by using H_2_O in the synthesis process, and then H_2_O captured in the network was evaporated at 80 °C for 24 h.

#### 4.3.2. Mechanical Properties

Mechanical tests were conducted with a Tuofeng tensile machine (TFW-5S). The ionogel samples were cut into dumbbell-like (50 mm × 4 mm × 2 mm) shapes for tensile performance measurements. The value of the stretching rate was set at 50 mm/min. The elastic modulus was measured from the slope of the tensile stress–tensile strain curve. The area of the tensile stress–tensile strain curve represents the toughness of the hydrogel.

#### 4.3.3. Conductivity

The resistance of the ionogel was determined using a Keithley 2450 source meter. The sample was a rectangular ionogel film (50 mm × 5 mm × 2 mm) sandwiched between two electrodes. The conductivity (*σ*) of the sample was computed by the formula: *σ* = *L*/(*S* × *R*), in which *L*, *S* and *R* are the length, the cross-section area and the resistance of the sample, respectively.

#### 4.3.4. Transparency

The transparency of the ionogel was characterized using an UV-visible spectrophotometer (PerkinElmer, lamada950, Waltham, MA, USA) in the wavelength range between 400 and 800 nm. The thickness of the sample was 2 mm.

#### 4.3.5. Thermal Analysis

The STA449C thermogravimetric analyzer (Netzsch, Waldkraiburg, Germany) was used to measure the thermal characteristics of the ionogel samples. The test condition was a temperature range of 30 to 600 °C and a scanning rate of 10 °C /min in an argon environment. The calorimetric information of the ionogel samples was observed using a differential scanning calorimeter (DSC250, TA Instruments, New Castle, DE, USA). The test condition was a temperature range of −90 to 50 °C and heating rate of 10 °C /min in a nitrogen environment.

#### 4.3.6. Adhesion Properties

The adhesion properties of the ionogels were evaluated by 90° peeling tests using the universal tensile machine. The ionogel sample (60 mm × 25 mm × 2 mm) was adhered on the surface of the substrate and then peeled off at a peeling speed of 50 mm/min. During the peeling process, a peeling direction of 90° was always maintained in the substrate. The applied substrate material included glass, plastic (PE film), stainless steel and copper to observe the adhesion properties of ionogel to the surface of different materials.

#### 4.3.7. Strain Sensing Properties

The TFW-5S tensile machine and the Keithley 2450 source meter were combined to measure resistance-change signals of the ionogel under different tensile strains. A DC voltage of 5 V was applied during the sensing process. The tensile machine was used to control the tensile speed and strain of the sample, and the source meter was applied to detect the variation of resistance of the sample according to the elongation strain. The gauge factor (GF) was calculated by the slope of relative resistance change versus strain. In order to prepare a flexible sensor, the ionogel film was cut into a strip (50 mm × 10 mm × 2 mm) and connected to the source meter by copper wire. The ionogel sensor was adhered to human skin to monitor human movement signals such as joint-bending, vocalization and walking gait. The relative resistance change was calculated by the formula ∆R/R_0_ (%) = |(R − R_0_)/R_0_| × 100, where R_0_ and R were the resistances of the original and stretched ionogel, respectively.

## Data Availability

The data presented in this study are available upon request from the corresponding author.

## Supplementary Materials

The following supporting information can be downloaded at: https://www.mdpi.com/article/10.3390/gels8120797/s1, Figure S1: curve-fitting analysis of FTIR spectra of the dried CS/PAA network and CS/PAA ionogel; Table S1: curve-fitting analysis result of the hydrogen-bonded functional groups; Table S2: dosage of the chemicals for different ionogel samples; Table S3: tensile strength, tensile elongation and toughness of different ionogel samples.
