# Supplementary material for: Highly Stretchable, Transparent and Adhesive Ionogel Based on Chitosan-Poly(acrylic acid) Double Networks for Flexible Strain Sensors
Source: Gels. 2022 Dec 5;8(12):797. doi: 10.3390/gels8120797 (PMC9777788; doi:10.3390/gels8120797)
Supplement: Supplementary file 1 [file gels-08-00797-s001.zip › gels-2060461-supplementary.pdf]

# supplementary

# Highly stretchable, transparent and adhesive ionogel based on chitosan-poly (acrylic acid) double networks for flexible strain sensor

Yuan Zhu, Xuemei Li, Zhenjie Zhao, Yongri Liang*, Li-Min Wang, and Ying Dan Liu*

State Key Lab of Metastable Materials Science and Technology, and College of Materials Science and Engineering, Yanshan University, Qinhuangdao 066004, P.R. China

Corresponding author email: liangyr@ysu.edu.cn (Y. Liang); ydliu@ysu.edu.cn (Y.D. Liu)

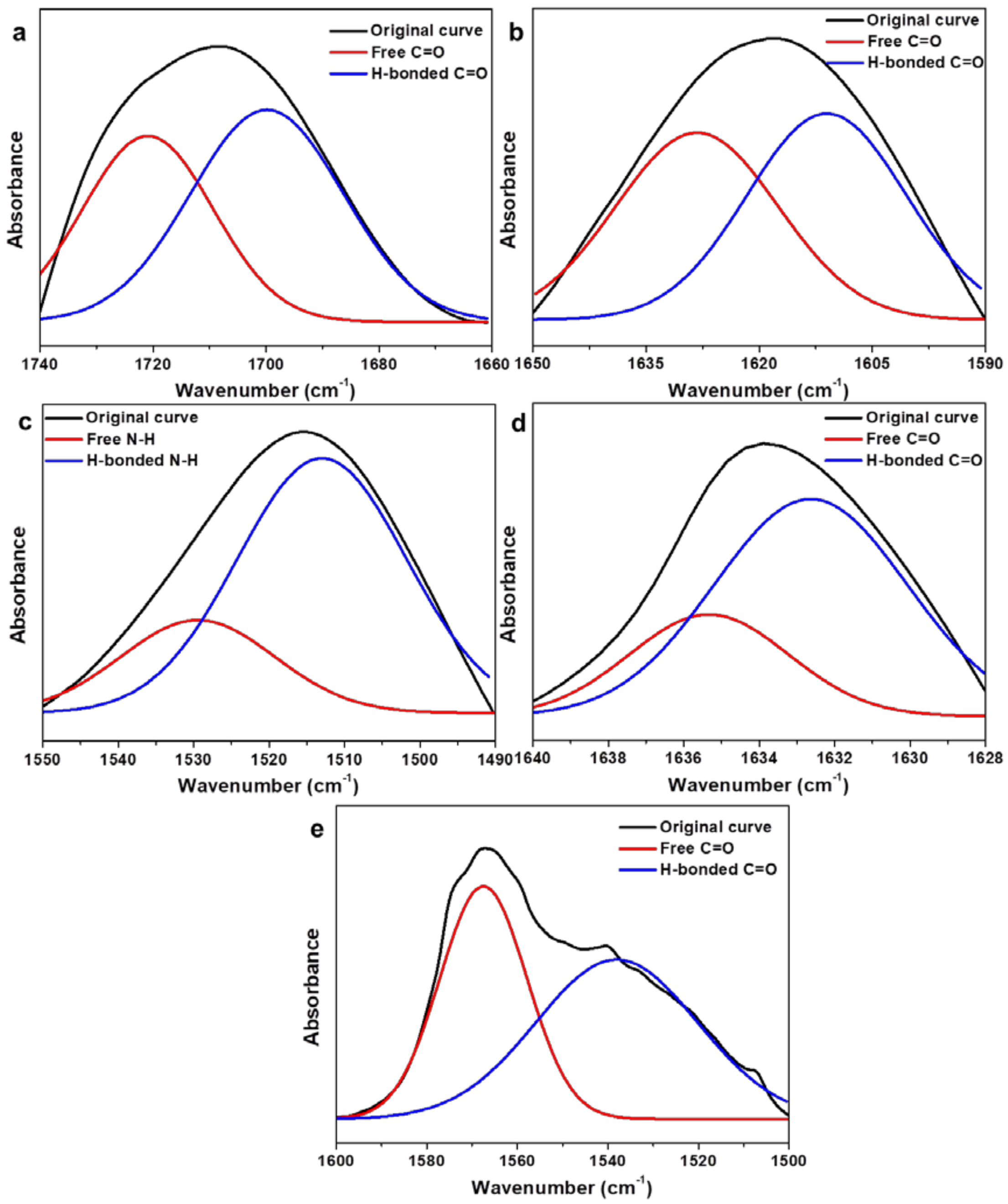


**Figure S1** Curve-fitting analysis of FTIR spectra of the C=O stretching region for CS (a) and PAA (b) of the dried CS/PAA network; the N-H stretching region (c) and the C=O stretching region (d) for CS in the CS/PAA ionogel, and the C=O stretching region for [EMIM][OAc] (e) in the CS/PAA ionogel.

**Table S1** Curve-fitting analysis result FTIR of hydrogen-bonded functional groups.

| **Functional group** | **Hydrogen-bonded functional groups** | **Free functional groups** | **The percentage of hydrogen-bonded functional groups (%)** |
|---|---|---|---|
| C=O of CS in CS/PAA | 0.744 | 0.535 | 58 |
| C=O of PAA in CS/PAA | 0.205 | 0.191 | 52 |
| N-H of CS in CS/PAA | 0.623 | 0.208 | 75 |
| C=O of CS in CS/PAA ionogel | 0.056 | 0.022 | 72 |
| C=O of [EMIM][OAc] in CS/PAA ionogel | 1.457 | 1.163 | 56 |

**Table S2** Dosage of the chemicals for different ionogel samples.

| **Sample** | **Contents of chemicals** | | | | |
|---|---|---|---|---|---|
| | **AA** | **[EMIM][OAc]** | **CS[a]** | **1173[a]** | **PEGDA[b]** |
| 1 | 40wt% | 60wt% | 0wt% | 1wt% | 0.025mol% |
| 2 | 60wt% | 40wt% | 5wt% | 1wt% | 0.025mol% |
| 3 | 50wt% | 50wt% | 5wt% | 1wt% | 0.025mol% |
| 4 | 40wt% | 60wt% | 5wt% | 1wt% | 0.025mol% |
| 5 | 35wt% | 65wt% | 5wt% | 1wt% | 0.025mol% |
| 6 | 30wt% | 70wt% | 5wt% | 1wt% | 0.025mol% |
| 7 | 40wt% | 60wt% | 1wt% | 1wt% | 0.05mol% |
| 8 | 40wt% | 60wt% | 3wt% | 1wt% | 0.05mol% |
| 9 | 40wt% | 60wt% | 5wt% | 1wt% | 0.05mol% |
| 10 | 40wt% | 60wt% | 7wt% | 1wt% | 0.05mol% |
| 11 | 40wt% | 60wt% | 5wt% | 1wt% | 0 mol% |
| 12 | 40wt% | 60wt% | 5wt% | 1wt% | 0.01mol% |
| 4 | 40wt% | 60wt% | 5wt% | 1wt% | 0.025mol% |
| 9 | 40wt% | 60wt% | 5wt% | 1wt% | 0.05mol% |
| 13 | 40wt% | 60wt% | 5wt% | 1wt% | 0.10mol% |

a The dosage of CS and 1173 is calculated as the weight percent of AA.

[b] The dosage of PEGDA is calculated as the molar percent of AA.

**Table S3** Tensile strength, tensile elongation and toughness of different ionogel samples.

| Sample | Tensile strength (MPa) | Tensile elongation (%) | Toughness ($MJ/m^3$) |
|---|---|---|---|
| 1 | 0.28 | 1556.53 | 1.44 |
| 2 | 5.42 | 144.32 | 5.99 |
| 3 | 4.31 | 720.75 | 12.03 |
| 4[a] | 2.04 | 1045.97 | 8.52 |
| 5 | 0.42 | 1494.99 | 3.36 |
| 6 | 0.14 | 370.80 | 0.35 |
| 7 | 0.65 | 1402.28 | 3.29 |
| 8[b] | 1.22 | 1157.40 | 5.13 |
| 9 | 2.18 | 963.19 | 8.61 |
| 10 | 1.22 | 662.14 | 4.15 |
| 11 | 0.91 | 968.74 | 4.78 |
| 12 | 1.45 | 935.58 | 5.43 |
| 13 | 0.36 | 615.65 | 3.55 |

a The sample for mechanical study and general analysis.

b The sample for extensive sensing performance study.
